# Book Review

**Published:** 1949-01

**Authors:** 


### Reviews of Books

**A Synopsis of Physiology.** By A. Rendle Short, B.Sc., M.D., F.R.C.S., C. L. G. Pratt, O.B.E., M.A., M.D., M.Sc., and C. C. N. Vass, M.Sc., Ph.D., M.B., Ch.B. Fourth Edition. Pp. x, 346. Illustrated. Bristol: John Wright & Sons Ltd. 1948. 20s.—It is difficult to praise this book, since Physiology cannot be satisfactorily presented in the form of a synopsis; yet some success has undoubtedly been achieved in condensing the subject-matter and bringing it reasonably up to date, so as to serve as a convenient aid in revision. It cannot take the place of a text-book. The ground is well covered, though there are some obvious omissions. Gout is not mentioned in the section on purine metabolism; and, as an example of the omission of important evidence, no reference is made to the results of cannulation of the capsule in the mammalian kidney or of other parts of the nephron.

On the whole, the balance is well preserved. A large amount of space is devoted to colour vision hypotheses. As might be expected in a book of this kind, the style leaves something to be desired. One particularly bad sentence reads : " Isletectomy may be obtained after pancreatectomy, or injection of extracts of anterior pituitary gland or alloxan." Here and there teleological statements appear where reasoned views, even if only brief, should be given. The format is handy, and the lay-out and printing are excellent ; the few diagrams are clear. There are several misprints which should be corrected in the next edition.

**Infra-red Irradiation.** By WILLIAM BEAUMONT, M.R.C.S., L.R.C.P. Third Edition. Pp. xii, 162. Illustrated. London : H. K. Lewis & Co. Ltd. 1948. 8s. 6d.—Students of physiotherapy will welcome this new edition of Dr. Beaumont's very readable little text-book on radiant heat. It has been considerably revised and the layout improved ; printing, paper and binding remain excellent. The physical basis, which many students find difficult, might be more fully explained. On pages 35 and 36 appear the remarkable statements that red rays are the shorter visible and blue rays the longer ! The correct description is on page 60. There are a few references to literature on the text pages, but a full bibliography would be a useful addition. The index is inadequate.

**Introduction to Diseases of the Chest.** By JAMES MAXWELL, M.D., F.R.C.P. Third Edition. Pp. xii, 307. Illustrated. London : Hodder & Stoughton Ltd. 1948. 12s. 6d.—The subject-matter of this book is well balanced, and the method of presentation is clear and concise, full use being made of paragraph headings and tabulation. The third edition has brought it up to date, particularly regarding penicillin therapy in respiratory infections. All these points commend it for the use of the student or practitioner, who desires fuller information on the subject than the average text-book gives. It is, therefore, the more unfortunate that the first complication of lobar pneumonia to be considered is lung abscess, and that for the treatment of " delayed resolution " it is thought necessary to suggest needling of the lung—a hazardous procedure at the best of times, especially if as an outside possibility a lung abscess were developing. There are, however, very few such points to detract from the soundness of the teaching in this most useful volume. A good selection of skiagrams is adequately reproduced at the end of the book, which is very moderately priced, considering the high standard of publication.

**The Background of Therapeutics.** By J. HAROLD BURN, M.D., F.R.C.S. Pp. vii, 367. Illustrated. London : Oxford University Press (Geoffrey Cumberlege). 1948. 22s. 6d.—Every now and again an outstanding book is published dealing with Clinical Medicine, a book that matters to every physician, to every general practitioner, and to every teacher in any branch of Medicine and Surgery. Professor Burn has chosen for discussion a few examples of the bearing which ascertained pharmacological fact has upon the practice of medicine and surgery. Teachers of the older generation stand at the bedside and speak as pundits to the young students, who often must know how out of date the pundits' pharmacology has become, and preserve a respectful but doubting silence. Here is a book for older clinicians to read with humility and the younger ones with apprehension, for, if they do not take care, their knowledge, too, will be found out of date.

**A Surgeon's Guide to Local Anæsthesia.** By C. E. CORLETTE, M.D., Ch.M., F.R.A.C.S. Pp. xi, 355. Illustrated. Bristol: John Wright & Sons Ltd. 1948. 30s.—It is a great pity that this excellent book is given the sub-title of " A Manual of Shockless Surgery," thus giving the impression that the author is prone to exaggeration ; it leads the reader to think that local anæsthesia is applicable only to those less severe operations in which the other factors which cause shock do not play a part, or that extravagant claims are being made. As a guide to the technique of local anæsthesia it is thorough and complete. In the early chapters there is a general review of the subject, and the reader is reminded of the dangers of spinal anæsthesia even when compared with chloroform. Stress is laid on the value of careful premedication, and on the use of adrenalin, and practical advice is given regarding the dangers of confusing various solutions. It is surprising that the need for an adequate syringe service using only syringes sterilized in an autoclave is not stressed. The actual technique of local anæsthesia is admirably described and well illustrated, and emphasizes the detailed knowledge of anatomy required. The anæsthesia devised for cystoscopy sounds complicated and might lead to trouble where there is infection of the urinary tract, and the use of the cautery for excision of hæmorrhoids is rarely employed nowadays. This book will with advantage be read by all surgeons, and should encourage those who rarely employ local anæsthesia to give it a wider trial. It can be recommended as an excellent guide to the technique.

